# JI017 Induces Cell Autophagy and Apoptosis via Elevated Levels of Reactive Oxygen Species in Human Lung Cancer Cells

**DOI:** 10.3390/ijms24087528

**Published:** 2023-04-19

**Authors:** Jin Mo Ku, Min Jeong Kim, Yu-Jeong Choi, Seo Yeon Lee, Ji-Yeong Im, Yong-Kyu Jo, Sanghoon Yoon, Ji-Hyun Kim, Jie Won Cha, Yong Cheol Shin, Seong-Gyu Ko

**Affiliations:** 1 Department of Preventive Medicine, College of Korean Medicine, Kyung Hee University, 1 Hoegi, Seoul 130-701, Republic of Korea; saory_ykm@naver.com (J.M.K.); syc99@khu.ac.kr (Y.C.S.); 2 Department of Science in Korean Medicine, Graduate School, Kyung Hee University, Seoul 130-701, Republic of Korea; jung8328@hanmail.net (M.J.K.); ehowlqk11@naver.com (Y.-J.C.); dltjdus0225@naver.com (S.Y.L.); 3 Department of Clinical Korean Medicine, Graduate School, Kyung Hee University, Seoul 130-701, Republic of Korea; jyhani@naver.com; 4 Department of Korean Medicine, Graduate School, Kyung Hee University, Seoul 130-701, Republic of Korea; ykcho0707@hanmail.net (Y.-K.J.); jhvoice@hanmail.net (J.-H.K.); 5 Department of Applied Korean Medicine, Graduate School, College of Korean Medicine, Kyung Hee University, Seoul 130-701, Republic of Korea; chin9yaaaa@gmail.com (S.Y.); goldfish310@hanmail.net (J.W.C.)

**Keywords:** autophagy, JI017, lung cancer, ROS

## Abstract

Lung cancer is one of the most common malignant tumors and a leading cause of cancer-related death in the worldwide. Various anticancer drugs, such as cisplatin and pemetrexed, have been developed for lung cancer treatment but due their drug resistance and side effects, novel treatments need to be developed. In this study, the efficacy of the natural drug JI017, which is known to have few side effects, was tested in lung cancer cells. JI017 inhibited A549, H460, and H1299 cell proliferation. JI017 induced apoptosis, regulated apoptotic molecules, and inhibited colony formation. Additionally, JI017 increased intracellular ROS generation. JI017 downregulated PI3K, AKT, and mTOR expression. JI017 increased the cytosolic accumulation of LC3. We found that JI017 promoted apoptosis through ROS-induced autophagy. Additionally, the xenograft tumor size was smaller in JI017-treated mice. We found that JI017 treatment increased MDA concentrations, decreased Ki-67 protein levels, and increased cleaved caspase-3 and LC3 levels in vivo. JI017 decreased cell proliferation and increased apoptosis by inducing autophagy signaling in H460 and H1299 lung cancer cells. Targeting JI017 and autophagy signaling could be useful in lung cancer treatment.

## 1. Introduction

Lung cancer is one of the most common malignant tumors and a leading cause of cancer-related death in the worldwide in both males and females, excluding sex-specific cancers [1]. Lung cancer is heterogenous in terms of pathological features: small-cell lung cancer (SCLC) accounts for ~14% of lung cancer patients and non-small cell lung cancer (NSCLC) accounts for ~82% of lung cancer patients [2,3]. The emergence of novel therapeutic methods has significantly improved the treatment of NSCLC, but the prognosis is still not good, and the overall 5-year survival rate of NSCLC patients is only 19.3% [4,5]. Various anticancer drugs, such as pemetrexed and cisplatin, have been used for the treatment of lung cancer, but these anticancer drugs cause acute kidney damage through nephrotoxicity and oxidative damage and cause side effects due to toxicity [6,7,8,9]. Therefore, the development of new therapeutic drugs with fewer side effects is necessary.

Autophagy is the process of removing damaged proteins and organelles within cells and recycling intracellular materials and energy, and this mechanism plays an important role in maintaining intracellular homeostasis [10,11,12,13,14]. Autophagy is activated in injury and disease in response to stresses such as nutrient deprivation, infection, and certain signaling pathways and contributes to cell survival [15,16]. Excessive autophagy is known to induce apoptosis [17]. For example, combined treatment with docetaxel and curcumin induces the apoptosis of malignant esophageal squamous cell carcinoma cells by increasing autophagy [18]. LC3 is a major participant in autophagy, and at initiation, the protein complex LC3-I is degraded to LC3-II, recruited into autophagosomes, and interacts with p62 [19]. Then, the formation of the lysosomal complex then leads to total proteolysis [20].

Reactive oxygen species (ROS) are highly reactive molecules formed from diatomic oxygen that are natural byproducts of normal oxygen metabolism, and they play an important role in homeostasis in the body [21]. Cellular stress caused by changes in the external environment dramatically increases intracellular ROS concentrations, which can cause severe damage to cellular structures, resulting in oxidative damage. Additionally, increased ROS levels are accompanied by apoptosis [22,23,24]. Therefore, ROS are an indicator of cellular stress and apoptosis [25,26]. Moreover, they are known to be important regulators of autophagy activation, and targeting increased autophagy and ROS production has been identified as a novel therapeutic approach for the treatment of several types of cancer [27,28].

Recently, studies on the anticancer effects of natural products have been conducted, and interest in natural product-derived medicines is increasing [29,30,31,32]. According to our previous report, *Angelica gigas* (*Ag*), *Zingiber officinale Roscoe* (*Zo*), and *Aconitum carmichaeli* (*Ac*) showed anticancer effects in the cell lines of several cancers, including brain, breast, prostate, colorectal, skin, and pancreatic cancer [33,34,35,36,37,38]. Furthermore, it is known that natural treatments for various inflammatory diseases and obesity have neuroprotective effects [39,40,41]. Decursin-inhibited tumor progression in head and neck squamous cell, as well as the active compound of *Ag*, induces apoptosis by inhibiting the PI3K–Akt axis in HeLa cells [42,43]. Among the active compounds of *Zo*, 6-gingerol suppresses tumor cell proliferation by blocking the nuclear translocation of HIF-1α in lung cancer [44].

Although the anticancer effects of JI017 are known, the efficacy of JI017 treatment for NSCLC has not yet been evaluated. In this study, we investigated whether JI017 exhibits NSCLC cell death and cell growth inhibitory effects. In addition, we analyzed the protein signaling pathway to elucidate the mechanism by which JI017 treats NSCLC.

## 2. Results

### 2.1. JI017-Induced Apoptosis and Inhibited Proliferation in Lung Cancer Cells

**Figure 1:**
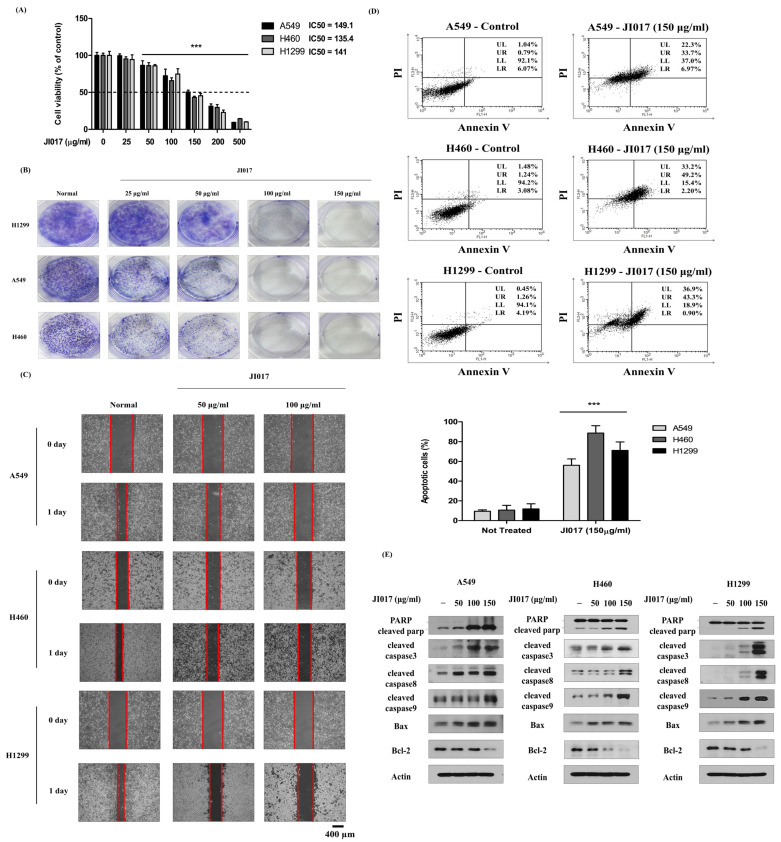
Effect of JI017 on A549, H460, and H1299 cell viability. (**A**) Cells were treated with different concentrations of JI017 for 24 h. (**B**) Cell viability was then measured using the MTS assay. H460, A549, and H1299 cells were treated with JI017 and exposed for 7 days, then the effect on cell growth was assessed using a colony formation assay. (**C**) The migration of JI017-treated cells was assessed using a wound-healing assay. Intervals between cells are marked with red lines. Scale bar = 400 µm. (**D**) H460 and H1299 cells were treated with JI017 for 24 h, stained with Annexin V/PI, and analyzed by flow cytometry. (**E**) Whole cell lysates were analyzed by Western blotting with anti-PARP; anti-cleaved caspase 3, -8, and -9; and anti-Bax, anti-Bcl-2, and anti-Actin antibodies. Data are presented as the mean ± SEM. *** *p* < 0.001 compared to untreated cells.

JI017 is known to be an effective anticancer drug for prostate, ovarian, and breast cancer [45,46,47,48]. Therefore, we investigated the effect of JI017 treatment on cell viability in several lung cancer cell lines. A549, H460, and H1299 cells were treated with different concentrations of JI017 for 24 h. Cell viability was then measured by MTS assay. We found that JI017 treatment significantly suppressed cell growth in a dose-dependent manner (Figure 1A). Additionally, JI017 treatment significantly reduced colony formation (Figure 1B). Moreover, cell migration was decreased in the JI017 treatment group compared to the control group (Figure 1C).

To investigate whether JI017 induces apoptosis, we performed an annexin V-FITC/PI assay in A549, H460, and H1299 cells. As expected, we found that the JI017 treatment group had an increased apoptosis rate in A549, H460, and H1299 cells, with apoptotic cell ratios of 9 to 56%, 10 to 88%, and 11 to 71%, respectively (Figure 1D). To confirm that caspase activation is induced by JI017 and is involved in apoptosis, we measured the expression of apoptotic molecules through Western blot analysis. We found that JI017 decreased the levels of Bcl-2 in A549, H460, and H1299 cells. Additionally, we found that JI017 increased the levels of Bax, cleaved caspase-3, cleaved caspase-8, cleaved caspase-9, and cleaved PARP in A549, H460, and H1299 cells (Figure 1E). These results confirmed that JI017 induced apoptosis through the apoptotic mechanisms of Bax, Bcl-2, caspase-3, caspase-8, and PARP in A549, H460, and H1299 cells.

### 2.2. JI017 Increased the Generation of Intracellular ROS

**Figure 2:**
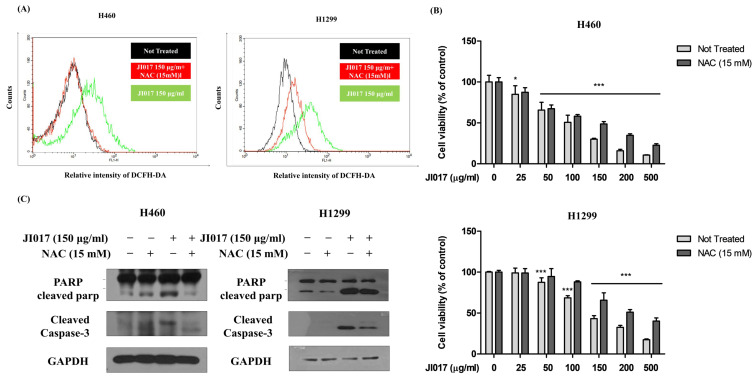
Effect of JI017 in A549, H460, and H1299 ROS accumulation. (**A**) Treatment with JI017 (150 μg/mL) in H460 and H1299 cells for 24 h; they were labeled with DCFH-DA (10 μM) for 30 min. Additionally, intracellular ROS levels were determined by flow cytometry. Pretreatment with NAC (15 mM) for 1 h was followed by treatment with various concentrations of JI017. (**B**) Cell viability was measured using the MTS assay. (**C**) Whole cell lysates were analyzed by Western blotting with anti-PARP, anti-cleaved caspase 3, and anti-GAPDH antibodies. Data are presented as the mean ± SEM. * *p* < 0.05 and *** *p* < 0.001 compared to untreated cells.

ROS is a small molecule, and it is primarily involved in several signaling pathways, and excessive ROS accumulation can induce lipid, nucleic acid, protein, and DNA damage and affect cancer cells to promote apoptosis [23,24]. We used the fluorescent dye 2′,7′-dichlorofluorescein diacetate (DCFH-DA) to measure ROS levels in lung cancer cells. The cells were treated with JI017 (150 μg/mL) or pretreatment with the ROS inhibitor NAC and JI017 for 24 h and labeled with DCFH-DA. The analysis measured signal intensity using a flow cytometer. JI017 treatment increased the DCFH-DA signal intensity in lung cancer cells. JI017 treatment combined with NAC pretreatment decreased the signal intensity of DCFH-DA in H460 and H1299 cells (Figure 2A). As a result, we confirmed that JI017 induced intracellular ROS generation. Additionally, we investigated whether ROS generated by JI017 mediated apoptosis. We added JI017 (150 μg/mL) to H460 and H1299 cells pretreated with NAC and performed MTS. JI017 treatment combined with NAC pretreatment decreased cell death was compared with that of JI017 treatment alone in H460 and H1299 cells (Figure 2B). Moreover, the cells treated with both JI017 and NAC showed decreased levels of the apoptosis markers cleaved caspase3 and cleaved PARP compared with those in the cells treated with JI017 alone (Figure 2C). These results suggested that JI017 induced apoptosis by increasing ROS production in lung cancer cells.

### 2.3. JI017-Induced Autophagy by Increasing LC3 Levels in H460 and H1299 Cells

**Figure 3:**
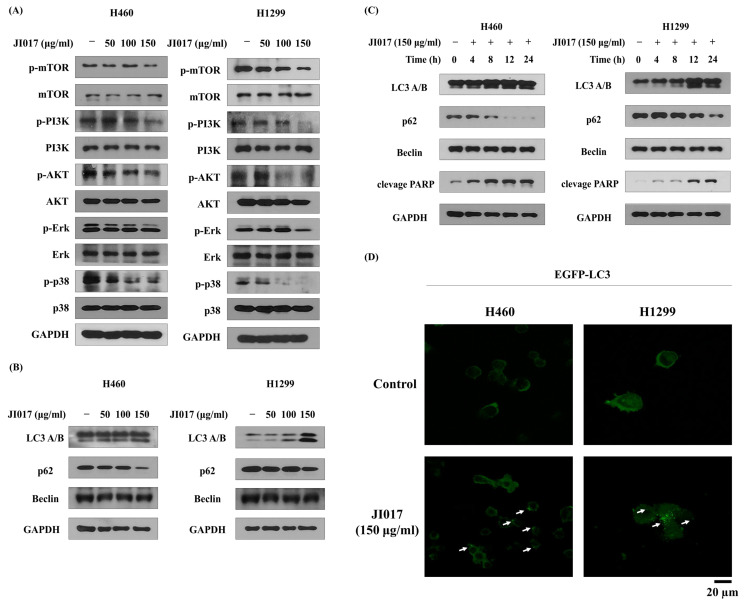
JI017 activated autophagy by suppressing the PI3K-AKT-mTOR pathway in H460 and H1299 cells. H460 and H1299 cells were treated with JI017 (50, 100, and 150 μg/mL) for 24 h. (**A**,**B**) Whole cell lysates were analyzed by Western blotting. (**C**) H460 and H1299 cells were treated with JI017 (150 ng/mL) for different times (4, 8, 12, and 24 h). (**D**) H460 and H1299 cells transfected with the pEGFP-LC3 vector were treated with JI017 (150 μg/mL) for 8 h. The Fluorescence microscopy analysis confirmed positive staining of LC3B puncta. LC3B puncta are indicated by white arrows. Scale bar = 20 µm.

We performed Western blot analysis to evaluate the expression of the PI3K-AKT-mTOR pathway and IF staining analysis to evaluate the expression of LC3. JI017 treatment decreased the levels of PI3K, AKT, and mTOR in H460 and H1299 cells (Figure 3A). The inhibition of mTOR is known to increase autophagy signaling [49]. To investigate whether JI017 induces autophagy, Western blot analysis was used, and it was confirmed in H460 and H1299 cells. The activation of the LC3 protein is a marker of autophagy, and we found that JI017 treatment increased the levels of LC3 A/B and p62 (Figure 3B). Additionally, the change in the LC3 level after JI017 treatment time was examined, and it was confirmed that both LC3 and cleaved PARP levels increased after 12 h of treatment with JI017 (Figure 3C). Additionally, the IF staining experiments showed that the cytoplasmic accumulation of LC3 was increased through JI017 treatment (Figure 3D). The autophagy pathway plays an important role in cancer cell death, and these results suggest that JI017 induces autophagy in lung cancer cells.

### 2.4. JI017 Induced Autophagy through Increased ROS Production in H460 and H1299 Cells

**Figure 4:**
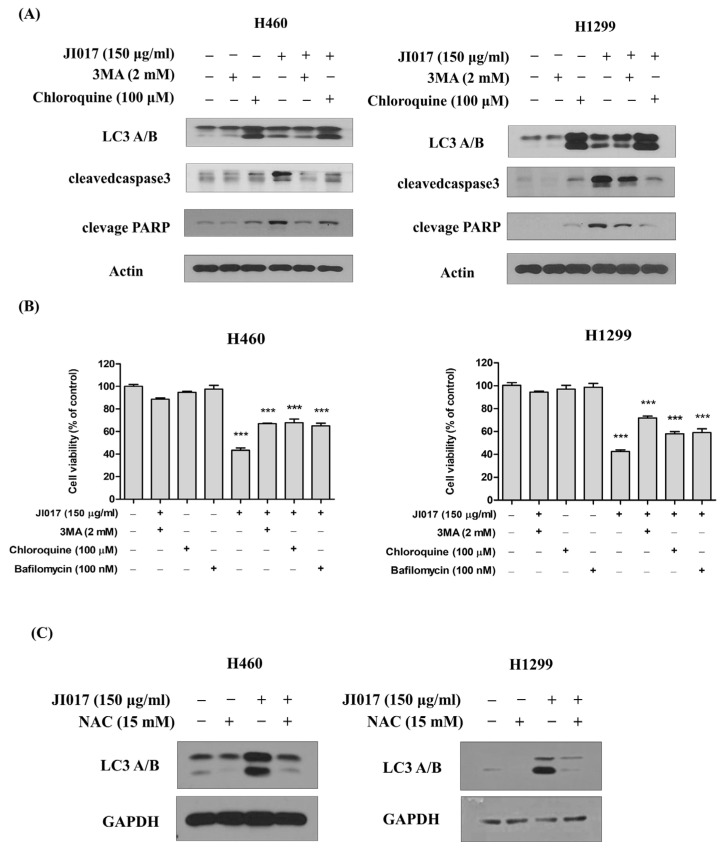
JI017 induced apoptosis through an autophagy pathway and ROS generation. (**A**) H460 and H1299 cells were treated with 3MA (2 mM) or chloroquine (100 μM) combined with JI017 (150 μg/mL). 3MA, chloroquine, or bafilomycin treatment was followed by treatment with JI017. (**B**) Cell viability was measured using MTS assay. (**C**) H460 and H1299 cells were treated with NAC (15 mM) and JI017 (150 μg/mL). Whole cell lysates were analyzed by Western blotting with anti-LC3 and anti-GAPDH antibodies. Data are presented as the mean ± SEM. *** *p* < 0.001 compared to untreated cells.

3MA is known to inhibit the autophagy pathway in the early stage, and chloroquine and Bafilomycin in the late stage [50,51,52]. Therefore, we treated H460 and H1299 cells with 3MA (2 mM) or chloroquine (100 μM) in combination with JI017 (150 μg/mL) and performed Western blot analysis. We found that 3MA treatment reduced the elevated LC3 levels induced by JI017 administration. Moreover, cells treated with JI017 and 3MA or chloroquine exhibited reduced levels of caspase3 cleavage and PARP cleavage compared to cells treated with JI017 alone, confirming reduced apoptosis (Figure 4A). To investigate whether JI017-induced apoptosis is regulated by autophagy inhibition, we compared cell viability after treatment with 3MA, bafilomycin, and chloroquine. Compared with JI017 treatment alone, JI017 treatment combined with 3MA or Bafilomycin or chloroquine decreased cell death by approximately 20% in H460 and H1299 cells (Figure 4B). Moreover, cells treated with JI017 combined with NAC decreased the level of LC3 compared with the cells treated with JI017 alone (Figure 2C). These results suggested that JI017 promoted apoptosis through ROS-induced autophagy in H460 and H1299 cells.

### 2.5. Effect of JI017 Suppressed Cell Growth In Vivo

**Figure 5:**
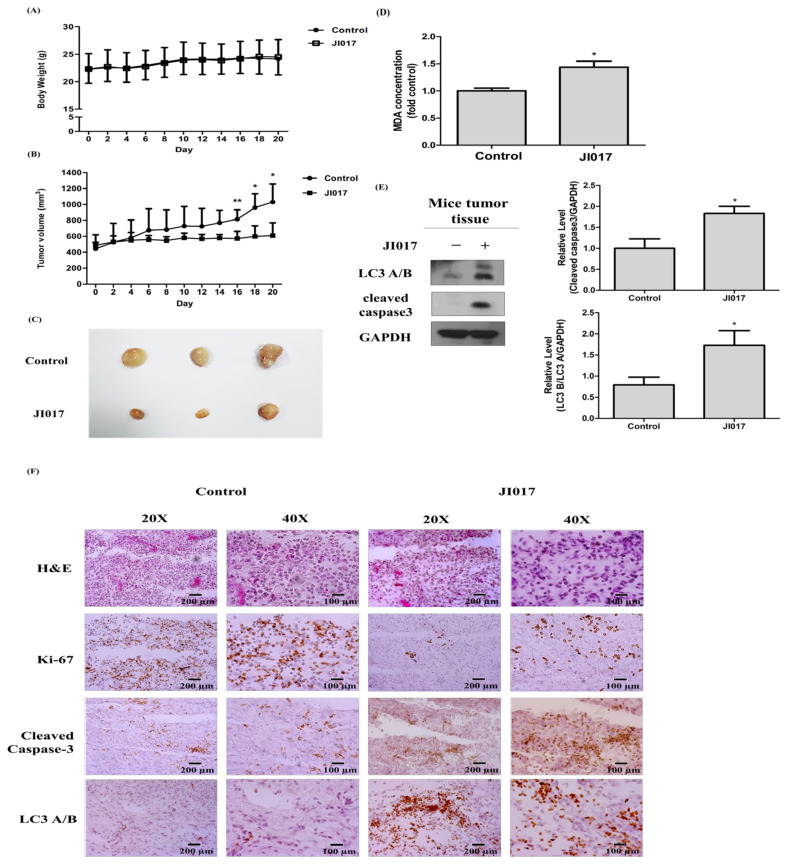
JI017 suppressed lung cancer cell growth in mice. BALB/c nude mice were subcutaneously injected with H460 cells. (**A**) The mouse body weight and (**B**) the tumor growth rate are shown. (**C**) Representative tumor images of the control group and the JI017-treated group. (**D**) The MDA concentration was assessed by a thiobarbituric reactive substance (TBARS) assay and normalized to the protein concentration. (**E**) Whole tissue lysates were analyzed by Western blotting with anti-LC3, anti-cleaved caspase 3, and anti-GAPDH antibodies. (**F**) The IHC staining of Ki-67 and cleaved caspase-3 and LC3 was carried out. Scale bar = 200 µm for 20× and Scale bar = 100 µm for 40×. The data are expressed as the mean ± SEM in all groups (*n* = 8–11). * *p* < 0.05 and ** *p* < 0.01 compared to the untreated group.

To further confirm the efficacy of JI017 for inhibiting cell growth in animal experiments, H460 cells were subcutaneously injected into nude mice. We did not find any change in body weight in the control group and the JI017-treated group (Figure 5A). The sizes of xenograft tumors in the JI017-treated mice were smaller compared to the xenograft tumors in the control mice, and the tumor suppression rate was 79.2%. These results indicated JI107 treatment slowed the tumor growth rate (Figure 5B,C). Because ROS reacts easily with lipids, free radical formation can be confirmed through lipid peroxidation markers. The peroxidation of membrane lipids can alter physical properties, such as lipid interaction, ion gradient, membrane fluidity, and permeability [53]. We found that JI017 treatment increased MDA concentrations in vivo (Figure 5D). Moreover, the addition of JI017 increased the levels of LC3 and cleaved caspase3 (Figure 5E). These results showed that the protein levels of Ki-67 decreased and the protein levels of cleaved caspase-3 and LC3 increased in the JI017-treated group compared to the control group (Figure 5F). Overall, the above results show that JI017 induced ROS in vivo and inhibited lung cancer cell proliferation and tumor growth.

## 3. Discussion

In this study, we found that treatment with JI017 in H460 and H1299 cells induced apoptosis by increasing autophagy pathway signaling. Although various anticancer drugs have been developed to treat lung cancer, new therapies are needed due to their many side effects. Recently, many studies have investigated anticancer effects through autophagy activity [54,55].

Additionally, interest in natural product-derived drugs is increasing in the pharmaceutical industry because of their low side effects [29,30,32]. Thus, our drug is a good suitable candidate for the treatment of human lung cancer.

It was reported that JI017 exerts anticancer effects and, thus, has potential as a new drug that death of cancer cells and inhibits cancer cell growth [45,48]. JI017 is a combination of *Ag*, *Zo*, and *Ac*, and its anti-cancer treatment effects have been reported through various mechanisms. Unlike simply confirming the active compound of a natural product, in a new compound, as a natural product complex, various effects are shown, and as a result, the treatment mechanism is multi-faceted, so there are still many areas to be studied. Therefore, we investigated the effect of JI017 treatment on lung cancer cells. We investigated whether JI017 treatment affected cell viability in H460 and H1299 cells and found that JI017 treatment significantly inhibited cell growth. In addition, growth inhibition was accompanied by the inhibition of cell migration and colony formation. We confirmed cancer cell apoptosis using flow cytometry after staining with annexin V/PI and analyzed protein changes in the apoptosis mechanism through Western blotting experiments. We found that JI017 treatment decreased the levels of Bcl-2 in A549, H460, and H1299 cells. Additionally, we found that the JI017 treatment of A549, H460 and H1299 cells increased the levels of Bax, cleaved caspase-3, cleaved caspase-8, cleaved caspase-9, and cleaved PARP, known as the markers of apoptosis. These results suggested that JI017 induced apoptosis in lung cancer cells.

Excessive ROS accumulation is known to promote apoptosis [56]. We evaluated the level of ROS in lung cancer cells using the fluorescent dye 2′,7′-dichlorofluorescein diacetate (DCFH-DA). We confirmed that JI017 treatment increased intracellular ROS generation. In addition, pretreatment with NAC, a ROS inhibitor, and treatment with JI017 decreased the rate of apoptosis, and we confirmed that the levels of caspase 3 and cleaved PARP, which are markers of apoptosis, were reduced in H460 and H1299 cells. These results suggested that JI017 induced apoptosis by ROS production in lung cancers.

**Figure 6:**
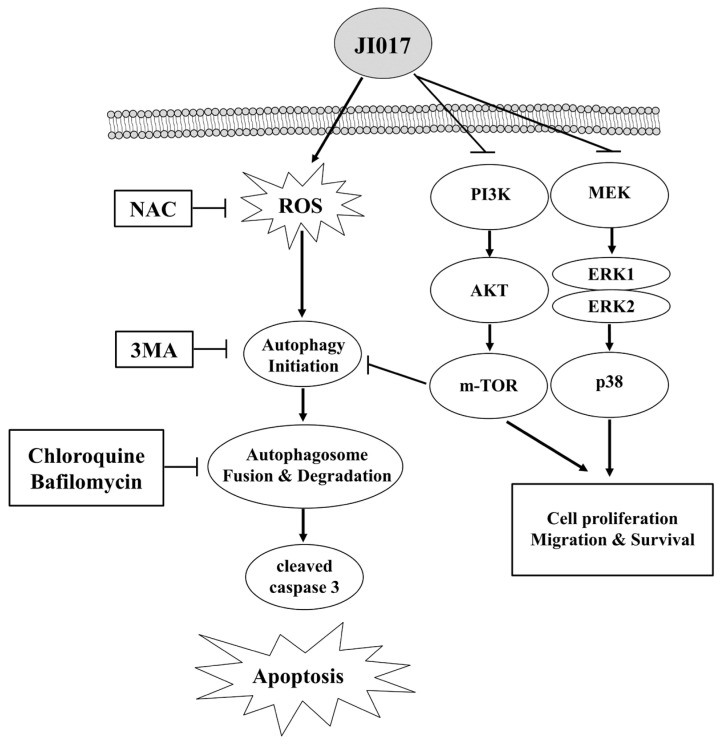
Mechanistic pathway diagram for the autophagy activation potency of JI017 through AKT, ERK inhibition, and ROS induction in lung cancer.

We investigated the pathway by which JI017 induced ROS accumulation and apoptosis in H460 and H1299 cells using Western blotting. The PI3K/Akt pathway is a representative regulator of growth, proliferation, the cell cycle, metastasis, apoptosis, and autophagy [57,58,59]. The inhibition of the PI3K/AKT/mTOR pathway can result in cell survival or death via autophagy or apoptosis, respectively [60,61,62,63,64]. JI017 decreased the levels of PI3K, AKT, and mTOR in H460 and H1299 cells. Therefore, we investigated whether JI017 induced autophagy using Western blot assay and IF staining analysis. We found that JI017 regulated LC3 A/B and p62 expression. The change in the LC3 level after JI017 treatment time was examined, and it was confirmed that the LC3 level and the cleavage of PARP increased after 12 h of treatment with JI017. Additionally, through IF staining results, it was confirmed that JI017 treatment increased the cytoplasmic accumulation of LC3 in cells. These results suggest that JI017 treatment induces autophagy and results in apoptosis in lung cancer. We treated JI017 with 3MA and chloroquine, which are autophagy inhibitors, to verify that JI017 induced apoptosis through the autophagy pathway. We found that 3MA reduced LC3 levels that increased with JI017 treatment. Moreover, cells treated with JI017 combined with 3MA or Bafilomycin or chloroquine showed decreased levels of apoptosis markers and cell death compared with those in cells treated with JI017 alone. In addition, cells treated with JI017 in combination with NAC exhibited reduced levels of LC3 compared to those in cells treated with JI017 alone, suggesting that JI017 promoted apoptosis through ROS-induced autophagy in H460 and H1299 cells (Figure 6). Moreover, we subcutaneously injected H460 cells into nude mice to confirm that JI017 has an inhibitory effect on lung cancer in vivo. Compared to control mice, the xenograft tumors in JI017-treated mice showed a lower growth rate and a TGI value of 79.2%. We found that JI017 treatment increased the MDA concentration in tumor tissues in vivo, and as a result, we determined that it induced ROS generation. Additionally, in the JI017 treatment group, the protein level of Ki-67 was decreased, and the protein levels of cleaved caspase-3 and LC3 were increased. Collectively, these findings suggested that JI017 inhibited lung cancer proliferation and tumor growth in vivo. JI017 clearly induced apoptosis in human lung cancer cells, making it a useful compound in the treatment of lung cancer. Additionally, targeting autophagy to treat lung cancer could be a useful therapeutic mechanism. Our study clearly demonstrates that the anticancer effect of JI017 in human lung cancer cells occurs through ROS-induced autophagy signaling. As a new natural compound anti-cancer treatment, we think that additional research on various mechanisms should be conducted.

## 4. Materials and Methods

### 4.1. Reagents

JI017 consists of *Ag*, *Ac*, and *Zo* components that were supplied by the Jaseng Hospital of Korean Medicine (Seoul, Republic of Korea). The roots were boiled for 3 h in distilled 70% ethanol. The extract was filtrated twice through Whatman grade 2 qualitative filter paper (GE Healthcare Life Sciences, Marlborough, MA, USA) to remove any insoluble materials. The filtrated extract was lyophilized to a powder using a freeze dryer (IlShinBioBase, Dongducheonsi, Gyeonggi, Republic of Korea) and stored at 4 °C. The dried extract was then dissolved in dimethyl sulfoxide (DMSO).

### 4.2. Cell Culture

A549, H460, and H1299 human lung cancer cells obtained from the American Type Culture Collection (ATCC) were maintained in RPMI 1640 or F-12K medium supplemented with 10% heat-inactivated fetal bovine serum (Invitrogen, Carlsbad, CA, USA) and 100 U/mL antibiotics–antimycotics (Invitrogen). Cells were maintained at 37 °C in a humidified incubator with 5% CO_2_.

### 4.3. Cell Viability Assay

An MTS assay was performed to determine cell viability. To accomplish this, cells (A549, H460, and H1299 cells) were seeded into a 96-well plate at a density of 3 × 10^3^ cells per well and treated 24 h later with varying concentrations of JI017 (25–500 μg/mL) for an additional 24 h. Ten microliters of MTS solution was added to each well of the plate, which was incubated in the dark at 37 °C for another 1 h 30 min. Optical density was measured at 450 nm using an ELISA plate reader (Versa Max, Molecular Devices, San Jose, CA, USA).

### 4.4. Flow Cytometric Analysis

Flow cytometry was used to analyze cell cycle distribution. Cells were seeded in 60 mm dishes. After 24 h, cells were cultured for an additional 24 h in the absence (control) or presence of JI017 (50–150 μg/mL). Trypsinized cells were washed with PBS and fixed in 95% ethanol containing 0.5% Tween-20 overnight at −20 °C. After washing with PBS, the cells were then incubated with 1 U/mL RNase A and 10 μg/mL PI for 30 min at room temperature in the dark. The DNA content in each cell nucleus was determined by a FACSCalibur flow cytometer (Becton-Dickinson, San Jose, CA, USA), and the cell cycle was analyzed using ModFit LT V2.0 software.

### 4.5. Annexin V-FITC Apoptosis Assay

Flow cytometry was used to analyze cell apoptosis. Cells were cultured in 60 mm dishes. After 24 h, cells were cultured for an additional 24 h in the absence (control) or presence of JI017 (50–150 μg/mL). Annexin V-FITC/PI double staining Apoptosis Detection Kit was purchased from Invitrogen (Waltham, MA, USA), and apoptosis assay was performed using a flow cytometer according to the manufacturer's instructions.

### 4.6. Colony Formation Assay

The cells were plated into 6-well culture plates at a density of 1 × 10^3^ cells/well. After 24 h, cells were cultured for an additional 10 d in the absence (control) or presence of JI017 (25, 50, 100, and 150 μg/mL) to allow colony formation. Colonies were stained with a 1% crystal violet solution (Amersco, Solon, OH, USA).

### 4.7. Western Blot Analysis

Cells were harvested, lysed with cell lysis buffer (50 mM Tris-Cl pH 7.4, 1% NP-40, 0.25% sodium deoxycholate, 0.1% SDS, 150 mM NaCl, 1 mM EDTA, and protease inhibitor) for 20 min and centrifuged at 13,000 rpm (4 °C) for 20 min. Twenty micrograms of protein were separated by SDS-polyacrylamide gel electrophoresis and transferred to a nitrocellulose membrane (Protran nitrocellulose membrane, Whatman, UK). The membrane was blocked with 5% nonfat milk, probed with specific primary antibodies, incubated with HRP-conjugated secondary IgG antibodies (Calbiochem, San Diego, CA, USA), and visualized using an enhanced chemiluminescence detection system (Amersham ECL kit, Amersham Pharmacia Biotech Inc., Piscataway, NJ, USA). Antibodies against cleaved caspase-8, -3, and -9; GAPDH; p38; phospho-Akt; phospho-mTOR; phospho-PI3K; and total Akt were obtained from Cell Signaling (Danvers, MA, USA). Antibodies against actin, Bax, Bcl-2, Beclin, PARP/p85, phospho-Erk, phospho-p38, total Erk, and total mTOR were obtained from Santa Cruz Biotechnology (Dallas, TX, USA). The anti-PI3K antibody was obtained from Merck Millipore (Burlington, MA, USA). The anti-LC3 antibody was obtained from Novus Biologicals (Centennial, CO, USA). The anti-p62 antibody was obtained from Abcam (Cambridge, UK).

### 4.8. Quantification of Autophagy

Autophagy was quantified by counting the percentage of cells showing the accumulation of GFP-LC3 in vacuoles. Cells presenting a mostly diffuse distribution of GFP-LC3 in the cytoplasm and nucleus were considered non-autophagic, whereas cells representing several intense punctate GFP-LC3 aggregates with no nuclear GFP-LC3 were classified as autophagic. Cells were fixed with paraformaldehyde (4% *w*/*v*) for GFP-LC3 and immunofluorescence assays. Images were acquired using confocal microscopy (Carl Zeiss, Oberkochen, Germany).

### 4.9. Lipid Peroxidation Measurement

Lipid peroxidation was measured using the Lipid Peroxidation (MDA) Assay Kit (Sigma-Aldrich, St. Louis, MO, USA) according to the manufacturer’s instructions. Extensive oxidative species generation can lead to the formation of malondialdehyde (MDA) as a result of the peroxidation of polyunsaturated fatty acids (PUFAs), which contain at least three double bonds. MDA can react with thiobarbituric acid (TBA) to form a colorimetric, as well a fluorescent, product, which is a well-known biomarker of the peroxidation of polyunsaturated lipids [65]. The tissues were lysed on ice using MDA lysis buffer containing 3 μL of butylated hydroxytoluene (BHT), and the samples were centrifuged at 13,000× *g* for 10 min. Next, a TBA solution was added and incubated at 95 °C for 60 min. The samples were chilled to room temperature in an ice bath for 10 min and transferred to a black bottomed 96-well plate. Optical density was measured at 532 nm using an ELISA plate reader.

### 4.10. Animal Studies

All animal experiment procedures were approved by the Kyung Hee University Institutional Animal Care and Use Committee (KHSASP-20-250). Five-week-old male BALB/C nu/nu mice were obtained from Raonbio (Seoul, Republic of Korea). The mice were maintained for 1 week under controlled temperature (23 ± 3 °C) and humidity (55 ± 15%) in a 12 h light/12 h dark cycle before initiating the experiment. Then, H460 cells were harvested and injected subcutaneously (5.0 × 10^6^ cells in 100 μL 1:1 PBS:Matrigel solution) into the right flank of the mice. When the xenografts reached a volume of 80–100 mm^3^, the animals were randomized into 2 groups (*n* = 6): the control group (saline) and the JI017 treatment group (500 mg/kg). The animals in the JI017 treatment group were treated with JI017 by oral administration every 2 days. Tumor sizes were measured every 2 days for changes in tumor growth, and tumor volumes were calculated using a standard formula: ½ (length × width^2^). Mice were sacrificed using carbon dioxide, followed by cervical dislocation, and the tumor tissue was isolated for further study, such as immunohistochemical (IHC) analyses.

### 4.11. IHC Analysis

To examine the protein expression of tumor-related genes in tumor tissue section samples, each serial frozen section was dried at RT for 20 min. After hydration, the samples were fixed with 4% paraformaldehyde and washed at 4 °C for 5 min. Then, the cells were blocked with bovine serum albumin (3% BSA) and probed with primary antibodies (1:100–1:400) overnight at 4 °C. Proteins were identified using anti-cleaved caspase3 and anti-LC3 and anti-Ki67 antibodies. The next day, the samples were washed and probed with secondary antibody for 30 min at RT. Then, the sections were incubated with Vectastain ABC reagent (Vector Laboratories, Inc., Burlingame, CA, USA, sk4100) for 30 min. Immune complexes were revealed via incubation with 3,3′-diaminobenzidine (DAB) at RT for 1 min based upon the targeted antigen. The sections were counterstained with hematoxylin and dehydrated on slides in 75%, 95%, and 100% ethanol for 1 min each, after which the sections were cleared in xylene for 5 min. Finally, the slides were mounted using mounting medium. Images were acquired using microscopy.

### 4.12. Statistical Analysis

All experimental data are expressed as the mean ± standard deviation (SD) or mean ± standard error of the mean (SEM) of at least three separate experiments. Statistical significance was determined using a one-way analysis of variance followed by the Tukey—Kramer multiple comparisons posttest to analyze differences between groups. A *p* value < 0.05 was considered to indicate a statistically significant difference, and *p* < 0.05, *p* < 0.01, and *p* < 0.001 are assigned separate symbols in the figures. All experiments were performed at least three times. All statistical analyses were performed using PRISM 5 software (GraphPad Software Inc., La Jolla, CA, USA).

## 5. Conclusions

In this study, we analyzed the effect of JI017 on human lung cancer cells. JI017 treatment induced the activation of autophagy signaling by increasing the generation of intracellular ROS. As a result, JI017 treatment decreased cell viability and caused apoptotic cell death in lung cancer cells. These results support JI017 as a favorable candidate drug for the treatment of human lung cancer.

## Data Availability

All data and materials are contained and described within the manuscript.

## Abbreviations

*Ac*, *Aconitum carmichaeli*; *Ag*, *Angelica gigas*; BHT, butylated hydroxytoluene; DAB, 3,3′-Diaminobenzidine; DMSO, dimethyl sulfoxide; EDTA, ethylenediaminetetraacetic acid; FACS, fluorescence-activated cell sorter; FBS, fetal bovine serum; GAPDH, glyceraldehyde 3-phosphate dehydrogenase; IHC, immunohistochemistry; MDA, malondialdehyde; NSCLC, non-small-cell lung cancer; PARP, poly (ADP-ribose) polymerase; PBS, phosphate-buffered saline; PI, propidium iodide; PMSF, phenylmethylsulfonyl fluoride; PUFAs, polyunsaturated fatty acids; ROS, reactive oxygen species; SCLC, small-cell lung cancer; SD, standard deviation; SDS, sodium dodecyl sulphate, TBA, thiobarbituric acid; *Zo*, *Zingiber officinale Roscoe*.

## References

[1] Goodman J.E., Mayfield D.B., Becker R.A., Hartigan S.B., Erraguntla N.K. (2020). Recommendations for further revisions to improve the International Agency for Research on Cancer (IARC) Monograph program. Regul. Toxicol. Pharmacol..

[2] Griffin R., Ramirez R.A. (2017). Molecular Targets in Non-Small Cell Lung Cancer. Ochsner J..

[3] Miller H.A., van Berkel V.H., Frieboes H.B. (2022). Lung cancer survival prediction and biomarker identification with an ensemble machine learning analysis of tumor core biopsy metabolomic data. Metab. Off. J. Metab. Soc..

[4] Iqbal N., Shukla N.K., Deo S.V., Agarwala S., Sharma D.N., Sharma M.C., Bakhshi S. (2016). Prognostic factors affecting survival in metastatic soft tissue sarcoma: An analysis of 110 patients. Clin. Transl. Oncol..

[5] Jonna S., Subramaniam D.S. (2019). Molecular diagnostics and targeted therapies in non-small cell lung cancer (NSCLC): An update. Discov. Med..

[6] Farooqui Z., Shahid F., Khan A.A., Khan F. (2017). Oral administration of Nigella sativa oil and thymoquinone attenuates long term cisplatin treatment induced toxicity and oxidative damage in rat kidney. Biomed. Pharmacother..

[7] Pierson-Marchandise M., Gras V., Moragny J., Micallef J., Gaboriau L., Picard S., Choukroun G., Masmoudi K., Liabeuf S., French National Network of Pharmacovigilance C. (2017). The drugs that mostly frequently induce acute kidney injury: A case—Noncase study of a pharmacovigilance database. Br. J. Clin. Pharmacol..

[8] Rombola G., Vaira F., Trezzi M., Chiappini N., Falqui V., Londrino F. (2015). Pemetrexed induced acute kidney injury in patients with non-small cell lung cancer: Reversible and chronic renal damage. J. Nephrol..

[9] Soni S., Basu M., Agrawal P., Kumar N., Bhatnagar A., Chhillar N. (2016). Multiple parametric approaches to assess acute radiation lung injury of rats radiation lung injury of rats. Ukr. Biochem. J..

[10] Li C., Zhang Y., Liu J., Kang R., Klionsky D.J., Tang D. (2021). Mitochondrial DNA stress triggers autophagy-dependent ferroptotic death. Autophagy.

[11] Li J., Liu J., Xu Y., Wu R., Chen X., Song X., Zeh H., Kang R., Klionsky D.J., Wang X. (2021). Tumor heterogeneity in autophagy-dependent ferroptosis. Autophagy.

[12] Li W., He P., Huang Y., Li Y.F., Lu J., Li M., Kurihara H., Luo Z., Meng T., Onishi M. (2021). Selective autophagy of intracellular organelles: Recent research advances. Theranostics.

[13] Kuma A., Hatano M., Matsui M., Yamamoto A., Nakaya H., Yoshimori T., Ohsumi Y., Tokuhisa T., Mizushima N. (2004). The role of autophagy during the early neonatal starvation period. Nature.

[14] Kim S.E., Overholtzer M. (2013). Autophagy proteins regulate cell engulfment mechanisms that participate in cancer. Semin. Cancer Biol..

[15] Chaudhary K., Shinde R., Liu H., Gnana-Prakasam J.P., Veeranan-Karmegam R., Huang L., Ravishankar B., Bradley J., Kvirkvelia N., McMenamin M. (2015). Amino acid metabolism inhibits antibody-driven kidney injury by inducing autophagy. J. Immunol..

[16] Jiang X., Overholtzer M., Thompson C.B. (2015). Autophagy in cellular metabolism and cancer. J. Clin. Investig..

[17] Chen C., Wang D., Yu Y., Zhao T., Min N., Wu Y., Kang L., Zhao Y., Du L., Zhang M. (2021). Legumain promotes tubular ferroptosis by facilitating chaperone-mediated autophagy of GPX4 in AKI. Cell Death Dis..

[18] Deng L., Wu X., Zhu X., Yu Z., Liu Z., Wang J., Zheng Y. (2021). Combination effect of curcumin with docetaxel on the PI3K/AKT/mTOR pathway to induce autophagy and apoptosis in esophageal squamous cell carcinoma. Am. J. Transl. Res..

[19] Galluzzi L., Green D.R. (2019). Autophagy-Independent Functions of the Autophagy Machinery. Cell.

[20] Tanida I., Ueno T., Kominami E. (2004). LC3 conjugation system in mammalian autophagy. Int. J. Biochem. Cell Biol..

[21] Whittington K., Harrison S.C., Williams K.M., Day J.L., McLaughlin E.A., Hull M.G., Ford W.C. (1999). Reactive oxygen species (ROS) production and the outcome of diagnostic tests of sperm function. Int. J. Androl..

[22] Ullah A., Ullah N., Nawaz T., Aziz T. (2023). Molecular Mechanisms of Sanguinarine in Cancer Prevention and Treatment. Anti-Cancer Agents Med. Chem..

[23] Joselin A.P., Hewitt S.J., Callaghan S.M., Kim R.H., Chung Y.H., Mak T.W., Shen J., Slack R.S., Park D.S. (2012). ROS-dependent regulation of Parkin and DJ-1 localization during oxidative stress in neurons. Hum. Mol. Genet..

[24] Mehterov N., Balazadeh S., Hille J., Toneva V., Mueller-Roeber B., Gechev T. (2012). Oxidative stress provokes distinct transcriptional responses in the stress-tolerant atr7 and stress-sensitive loh2 Arabidopsis thaliana mutants as revealed by multi-parallel quantitative real-time PCR analysis of ROS marker and antioxidant genes. Plant Physiol. Biochem..

[25] Shin D.Y., Kim G.Y., Li W., Choi B.T., Kim N.D., Kang H.S., Choi Y.H. (2009). Implication of intracellular ROS formation, caspase-3 activation and Egr-1 induction in platycodon D-induced apoptosis of U937 human leukemia cells. Biomed. Pharmacother..

[26] Ullah A., Leong S.W., Wang J., Wu Q., Ghauri M.A., Sarwar A., Su Q., Zhang Y. (2021). Cephalomannine inhibits hypoxia-induced cellular function via the suppression of APEX1/HIF-1alpha interaction in lung cancer. Cell Death Dis..

[27] Wang L., Fu X., Hyun J., Xu J., Gao X., Jeon Y.J. (2023). In Vitro and In Vivo Protective Effects of Agaro-Oligosaccharides against Hydrogen Peroxide-Stimulated Oxidative Stress. Polymers.

[28] Yang H.L., Lin Y.A., Pandey S., Liao J.W., Way T.D., Yeh Y.L., Chen S.J., Hseu Y.C. (2022). In vitro and in vivo anti-tumor activity of Antrodia salmonea against twist-overexpressing HNSCC cells: Induction of ROS-mediated autophagic and apoptotic cell death. Food Chem. Toxicol..

[29] Hafeez B.B., Siddiqui I.A., Asim M., Malik A., Afaq F., Adhami V.M., Saleem M., Din M., Mukhtar H. (2008). A dietary anthocyanidin delphinidin induces apoptosis of human prostate cancer PC3 cells in vitro and in vivo: Involvement of nuclear factor-kappaB signaling. Cancer Res..

[30] Hafeez B.B., Mustafa A., Fischer J.W., Singh A., Zhong W., Shekhani M.O., Meske L., Havighurst T., Kim K., Verma A.K. (2014). alpha-Mangostin: A dietary antioxidant derived from the pericarp of *Garcinia mangostana* L. inhibits pancreatic tumor growth in xenograft mouse model. Antioxid. Redox Signal..

[31] Hafeez B.B., Fischer J.W., Singh A., Zhong W., Mustafa A., Meske L., Sheikhani M.O., Verma A.K. (2015). Plumbagin Inhibits Prostate Carcinogenesis in Intact and Castrated PTEN Knockout Mice via Targeting PKCepsilon, Stat3, and Epithelial-to-Mesenchymal Transition Markers. Cancer Prev. Res..

[32] Lall R.K., Adhami V.M., Mukhtar H. (2016). Dietary flavonoid fisetin for cancer prevention and treatment. Mol. Nutr. Food Res..

[33] Choi H.S., Cho S.G., Kim M.K., Kim M.S., Moon S.H., Kim I.H., Ko S.G. (2016). Decursin in Angelica gigas Nakai (AGN) Enhances Doxorubicin Chemosensitivity in NCI/ADR-RES Ovarian Cancer Cells via Inhibition of P-glycoprotein Expression. Phytother. Res..

[34] Li J.J., Chen W.L., Wang J.Y., Hu Q.W., Sun Z.P., Zhang S., Liu S., Han X.H. (2017). Wenshen Zhuanggu formula effectively suppresses breast cancer bone metastases in a mouse Xenograft model. Acta Pharmacol. Sin..

[35] Nedungadi D., Binoy A., Vinod V., Vanuopadath M., Nair S.S., Nair B.G., Mishra N. (2021). Ginger extract activates caspase independent paraptosis in cancer cells via ER stress, mitochondrial dysfunction, AIF translocation and DNA damage. Nutr. Cancer.

[36] Kweon B., Han Y.H., Kee J.Y., Mun J.G., Jeon H.D., Yoon D.H., Choi B.M., Hong S.H. (2020). Effect of Angelica gigas Nakai Ethanol Extract and Decursin on Human Pancreatic Cancer Cells. Molecules.

[37] Hamza A.A., Heeba G.H., Hamza S., Abdalla A., Amin A. (2021). Standardized extract of ginger ameliorates liver cancer by reducing proliferation and inducing apoptosis through inhibition oxidative stress/ inflammation pathway. Biomed. Pharmacother..

[38] Nemoto Y., Satoh K., Toriizuka K., Hirai Y., Tobe T., Sakagami H., Nakashima H., Ida Y. (2002). Cytotoxic and radical scavenging activity of blended herbal extracts. In Vivo.

[39] Semwal R.B., Semwal D.K., Combrinck S., Viljoen A.M. (2015). Gingerols and shogaols: Important nutraceutical principles from ginger. Phytochemistry.

[40] Zhou G., Tang L., Zhou X., Wang T., Kou Z., Wang Z. (2015). A review on phytochemistry and pharmacological activities of the processed lateral root of Aconitum carmichaelii Debeaux. J. Ethnopharmacol..

[41] Sowndhararajan K., Kim S. (2017). Neuroprotective and Cognitive Enhancement Potentials of Angelica gigas Nakai Root: A Review. Sci. Pharm..

[42] Zhu M.L., Li J.C., Wang L., Zhong X., Zhang Y.W., Tan R.Z., Wang H.L., Fan J.M., Wang L. (2021). Decursin inhibits the growth of HeLa cervical cancer cells through PI3K/Akt signaling. J. Asian Nat. Prod. Res..

[43] Joo M., Heo J.B., Kim S., Kim N., Jeon H.J., An Y., Song G.Y., Kim J.M., Lee H.J. (2022). Decursin inhibits tumor progression in head and neck squamous cell carcinoma by downregulating CXCR7 expression in vitro. Oncol. Rep..

[44] Kim M.J., Ku J.M., Choi Y.J., Lee S.Y., Hong S.H., Kim H.I., Shin Y.C., Ko S.G. (2022). Reduced HIF-1alpha Stability Induced by 6-Gingerol Inhibits Lung Cancer Growth through the Induction of Cell Death. Molecules.

[45] Kim T.W., Ko S.G. (2021). The Herbal Formula JI017 Induces ER Stress via Nox4 in Breast Cancer Cells. Antioxidants.

[46] Kim T., Ko S.G. (2021). JI017, a Complex Herbal Medication, Induces Apoptosis via the Nox4-PERK-CHOP Axis in Ovarian Cancer Cells. Int. J. Mol. Sci..

[47] Lee J.H., Ji H., Ko S.G., Kim W. (2021). JI017 Attenuates Oxaliplatin-Induced Cold Allodynia via Spinal TRPV1 and Astrocytes Inhibition in Mice. Int. J. Mol. Sci..

[48] Kim M.J., Ku J.M., Hong S.H., Kim H.I., Kwon Y.Y., Park J.S., Jung D.H., Shin Y.C., Ko S.G. (2021). In vitro Anticancer Effects of JI017 on Two Prostate Cancer Cell Lines Involve Endoplasmic Reticulum Stress Mediated by Elevated Levels of Reactive Oxygen Species. Front. Pharmacol..

[49] Wang R., Ha K.Y., Dhandapani S., Kim Y.J. (2022). Biologically synthesized black ginger-selenium nanoparticle induces apoptosis and autophagy of AGS gastric cancer cells by suppressing the PI3K/Akt/mTOR signaling pathway. J. Nanobiotechnol..

[50] Vlahos C.J., Matter W.F., Hui K.Y., Brown R.F. (1994). A specific inhibitor of phosphatidylinositol 3-kinase, 2-(4-morpholinyl)-8-phenyl-4H-1-benzopyran-4-one (LY294002). J. Biol. Chem..

[51] Powis G., Bonjouklian R., Berggren M.M., Gallegos A., Abraham R., Ashendel C., Zalkow L., Matter W.F., Dodge J., Grindey G. (1994). Wortmannin, a potent and selective inhibitor of phosphatidylinositol-3-kinase. Cancer Res..

[52] Blommaart E.F., Krause U., Schellens J.P., Vreeling-Sindelarova H., Meijer A.J. (1997). The phosphatidylinositol 3-kinase inhibitors wortmannin and LY294002 inhibit autophagy in isolated rat hepatocytes. Eur. J. Biochem..

[53] Gaschler M.M., Stockwell B.R. (2017). Lipid peroxidation in cell death. Biochem. Biophys. Res. Commun..

[54] Huang J., Ye Y., Xiao Y., Ren Q., Zhou Q., Zhong M., Jiao L., Wu L. (2022). Geniposide ameliorates glucocorticoid-induced osteoblast apoptosis by activating autophagy. Biomed. Pharmacother..

[55] Kong W., Zhu H., Zheng S., Yin G., Yu P., Shan Y., Liu X., Ying R., Zhu H., Ma S. (2022). Larotrectinib induces autophagic cell death through AMPK/mTOR signalling in colon cancer. J. Cell. Mol. Med..

[56] She Y.Y., Lin J.J., Su J.H., Chang T.S., Wu Y.J. (2022). 4-Carbomethoxyl-10-Epigyrosanoldie E Extracted from Cultured Soft Coral Sinularia sandensis Induced Apoptosis and Autophagy via ROS and Mitochondrial Dysfunction and ER Stress in Oral Cancer Cells. Oxid. Med. Cell. Longev..

[57] Alzahrani A.S. (2019). PI3K/Akt/mTOR inhibitors in cancer: At the bench and bedside. Semin. Cancer Biol..

[58] Pompura S.L., Dominguez-Villar M. (2018). The PI3K/AKT signaling pathway in regulatory T-cell development, stability, and function. J. Leukoc. Biol..

[59] Li H., Prever L., Hirsch E., Gulluni F. (2021). Targeting PI3K/AKT/mTOR Signaling Pathway in Breast Cancer. Cancers.

[60] Fang S., Wan X., Zou X., Sun S., Hao X., Liang C., Zhang Z., Zhang F., Sun B., Li H. (2021). Arsenic trioxide induces macrophage autophagy and atheroprotection by regulating ROS-dependent TFEB nuclear translocation and AKT/mTOR pathway. Cell Death Dis..

[61] Han X., Zhong Z., Kou J., Zheng Y., Liu Z., Jiang Y., Zhang Z., Gao Z., Cong L., Tian Y. (2018). ROS Generated by Upconversion Nanoparticle-Mediated Photodynamic Therapy Induces Autophagy Via PI3K/AKT/ mTOR Signaling Pathway in M1 Peritoneal Macrophage. Cell. Physiol. Biochem..

[62] Zhao J., Sun Y., Shi P., Dong J.N., Zuo L.G., Wang H.G., Gong J.F., Li Y., Gu L.L., Li N. (2015). Celastrol ameliorates experimental colitis in IL-10 deficient mice via the up-regulation of autophagy. Int. Immunopharmacol..

[63] Lee H.W., Jang K.S., Choi H.J., Jo A., Cheong J.H., Chun K.H. (2014). Celastrol inhibits gastric cancer growth by induction of apoptosis and autophagy. BMB Rep..

[64] Guertin D.A., Sabatini D.M. (2007). Defining the role of mTOR in cancer. Cancer Cell.

[65] Gueraud F., Atalay M., Bresgen N., Cipak A., Eckl P.M., Huc L., Jouanin I., Siems W., Uchida K. (2010). Chemistry and biochemistry of lipid peroxidation products. Free Radic. Res..

